# Distribution and Evolutionary History of Sialic Acid Catabolism in the Phylum *Actinobacteria*

**DOI:** 10.1128/spectrum.02380-21

**Published:** 2022-01-12

**Authors:** Yisong Li, Ying Huang

**Affiliations:** a State Key Laboratory of Microbial Resources, Institute of Microbiology, Chinese Academy of Sciences, Beijing, China; b College of Life Sciences, University of Chinese Academy of Sciences, Beijing, China; c Microbial Technology Institute and State Key Laboratory of Microbial Technology, Shandong University, Qingdao, China; South China Sea Institute of Oceanology, Chinese Academy of Sciences

**Keywords:** sialic acid catabolism, *Actinobacteria*, evolution, distribution, horizontal gene transfer, free-living species, environmental adaptation

## Abstract

Sialic acids are present in humans and other metazoans, playing essential roles in physiological and pathological processes. Commensal and pathogenic bacteria have evolved the capacity to utilize sialic acids as nutrient and energy sources. However, in some actinobacteria, sialic acid catabolism (SAC) is associated with free-living populations. To unravel the distribution and evolutionary history of SAC in the phylum *Actinobacteria*, we analyzed the presence and diversity of the putative SAC gene cluster (*nan*) in 7,180 high-quality, nonredundant actinobacterial genomes that covered 1,969 species. The results showed that ∼13% of actinobacterial species had the potential to utilize sialic acids, with 45 species capable of anhydro-SAC, all except two of them through the canonical pathway. These species belonged to 20 orders and 81 genera, with ∼36% of them from four genera, *Actinomyces*, *Bifidobacterium*, *Corynebacterium*, and *Streptomyces*. Moreover, ∼40% of the *nan*-positive species are free living. Phylogenetic analysis of the key *nan* genes, *nanA*, *nanK*, and *nanE*, revealed a strong signal of horizontal gene transfer (HGT), accompanied with vertical inheritance and gene loss. This evolutionary pattern led to high diversity and differential distribution of *nan* among actinobacterial taxa and might cause the cluster to spread to some free-living species while losing in some host-associated species. The evolution of SAC in actinobacteria probably represents the evolution of certain kinds of noncore bacterial functions for environmental adaptation and lifestyle switch, in which HGT plays a dominant role.

**IMPORTANCE** Sialic acids play essential roles in the physiology of humans and other metazoan animals, and microbial sialic acid catabolism (SAC) is one of the processes critical for pathogenesis. To date, microbial SAC is studied mainly in commensals and pathogens, while its distribution in free-living microbes and evolutionary pathway remain largely unexplored. Here, by examining all actinobacterial genomes available, we demonstrate that putative SAC is present in a small proportion of actinobacterial species, of which, however, ∼40% are free-living species. We also reveal remarkable difference in the distribution of SAC among actinobacterial taxa and high diversity of the putative SAC gene clusters. HGT plays a significant role in the evolution of SAC, accompanied with vertical inheritance and gene loss. Our results provide a comprehensive and systematic picture of the distribution and evolutionary history of SAC in actinobacteria, expanding the current knowledge on bacterial adaptation and diversification.

## INTRODUCTION

Sialic acids, or neuraminic acids, is the designation given to a family of nine-carbon keto sugar acids, the most common of which is N-acetyl-neuraminic acid (Neu5Ac) (1). Sialic acids are generally found at the outermost end of glycan chains present in glycoconjugates, which decorate cell surfaces and mediate a diverse range of cell-cell or cell-molecule interactions in eukaryotes (2, 3). Sialic acids are also major components of mucin (4–6), modulating a wide variety of physiological and pathological processes and playing pivotal roles in complex metazoan animals (7, 8). Because of the extensive distribution of sialic acids in metazoans, commensal and pathogenic bacteria have evolved the capacity to utilize sialic acids as carbon, nitrogen, and energy sources (2, 9, 10). Previous studies have shown that sialic acid catabolism (SAC) among bacteria is confined mainly to commensals and pathogens (9, 11–13). In addition, considering the important function of sialic acids for vertebrates, microbial SAC has been regarded as a virulence determinant in a range of infectious diseases (2, 9). Therefore, it is of great significance to investigate the distribution and evolution of SAC in microorganisms.

The complete SAC pathway usually contains the following steps. First, sialidases cleave terminal Neu5Ac residues from host glycoconjugates (14, 15). There are many different sialidases across microbes. Those that catalyze sialic acid cleavage can be separated into two classes: hydrolytic sialidases that release Neu5Ac and intramolecular *trans*-sialidases (IT-sialidases) that release 2,7-anhydro-Neu5Ac (16). Then, free (2,7-anhydro-)Neu5Ac enters into cells via four specialized transport systems: the major facilitator superfamily (MFS) permease, the tripartite ATP-independent periplasmic (TRAP) transporter, the sodium solute symporter (SSS), and the ATP-binding cassette (ABC) transporter (17). According to their evolutionary origins, these transporters can be further classified into eight sialic acid transporter (ST) families (ST1 to ST8) (18). After transport, 2,7-anhydro-Neu5Ac is converted back into Neu5Ac by cytoplasmic oxidoreductase NanOx (NanY in Escherichia coli) (19) and intracellular sialic acid is broken down to *N*-acetylglucosamine-6-phosphate (GlcNAc-6-P) by two alternative pathways. The canonical (Escherichia coli paradigm) pathway is composed of the sequential action of Neu5Ac lyase (NanA), N-acetyl-mannosamine kinase (NanK), and N-acetyl-mannosamine epimerase (NanE) (20), and the *Bacteroidetes* paradigm pathway relies on NanA, NanE-II (GlcNAc epimerase), and RokA (glucokinase) (21, 22). Finally, GlcNAc-6-P is deacetylated by GlcNAc-6-P deacetylase (NagA) and further converted by glucosamine-6-P deaminase (NagB) into fructose-6-phosphate, which enters into the subsequent glucolytic pathway (2, 11). In bacteria, the genes involved in the first three intracellular steps of the canonical SAC pathway or the first two steps of the *Bacteroidetes* pathway usually cluster together, forming a gene cluster denominated as *nan* (15, 23). However, *nagA* and *nagB* vary a lot in their locations among different genomes encoding SAC, either within or far away from the *nan* cluster, and the sialidase gene is not necessary for all strains capable of SAC (9, 15, 24). For ease of analysis, a complete canonical SAC system is usually defined as one that minimally includes clustered *nanA*, *nanK*, and *nanE* (2, 23), and an alternative *Bacteroidetes* SAC system minimally includes clustered *nanA* and *nanE-II* and contains *rokA*, too (21, 22).

To date, our understanding of SAC comes largely from commensal and pathogenic bacteria, biasing the knowledge of its ecology and evolution. *Actinobacteria* is a phylum of ubiquitous Gram-positive bacteria with high genomic G+C contents and diverse physiological and metabolic properties. The phylum has been attracting much attention as a rich source of various bioactive substances and for the complex evolution and diversification processes (25, 26). Although members of this phylum are widely distributed in both terrestrial and aquatic ecosystems, they also contain a large number of pathogens and commensals of humans and animals, such as the genera *Actinomyces* and *Bifidobacterium* (27, 28). It has been proved that some actinobacteria have evolved the capacity to degrade sialic acids (14, 29). In particular, there is experimental evidence showing that some free-living actinobacterial species can utilize sialic acids as a sole carbon and energy source. For example, Corynebacterium glutamicum can grow on Neu5Ac and has a full complement of genes for SAC, suggesting that SAC may have physiological roles in the soil environment (30). Our recent studies on different *Streptomyces* species, Streptomyces albidoflavus and Streptomyces olivaceus, residing in both free-living and insect-associated habitats have demonstrated that SAC is significantly associated, however, with free-living strains (31, 32). Despite these, the phylogenetic and ecological distribution and evolutionary pattern of SAC in the phylum *Actinobacteria* remain uncharacterized. Based on the NanA phylogeny and transporter distribution, McDonald et al. showed that SAC has evolved multiple times in bacteria but all strains of *Actinobacteria* form a supercluster that is the most divergent from other bacteria (except that the authors mistakenly assigned *Actinobacillus* of the phylum *Proteobacteria* and *Mycoplasma* of the phylum *Tenericutes* to *Actinobacteria*) (13). This likely implies that the evolution of SAC within actinobacteria is relatively independent from other bacteria, thus making *Actinobacteria* an ideal sample for studying how SAC spreads and evolves within an ancient bacterial phylum.

Here, we surveyed all available genomes of *Actinobacteria* to characterize the distribution and evolutionary history of putative SAC in the phylum. We assessed the presence/absence of SAC genes and gene clusters among actinobacterial taxa at different taxonomic levels and among both free-living and host-associated species. In addition, we characterized the phylogeny and genetic organization of the *nan* clusters. The study provides the first systematic and comprehensive insights into the diversity and evolutionary mechanism of actinobacterial SAC and expands the current knowledge of the genetic basis of bacterial adaptation and diversity.

## RESULTS

### Distribution of putative SAC genes in *Actinobacteria*.

**FIG 1:**
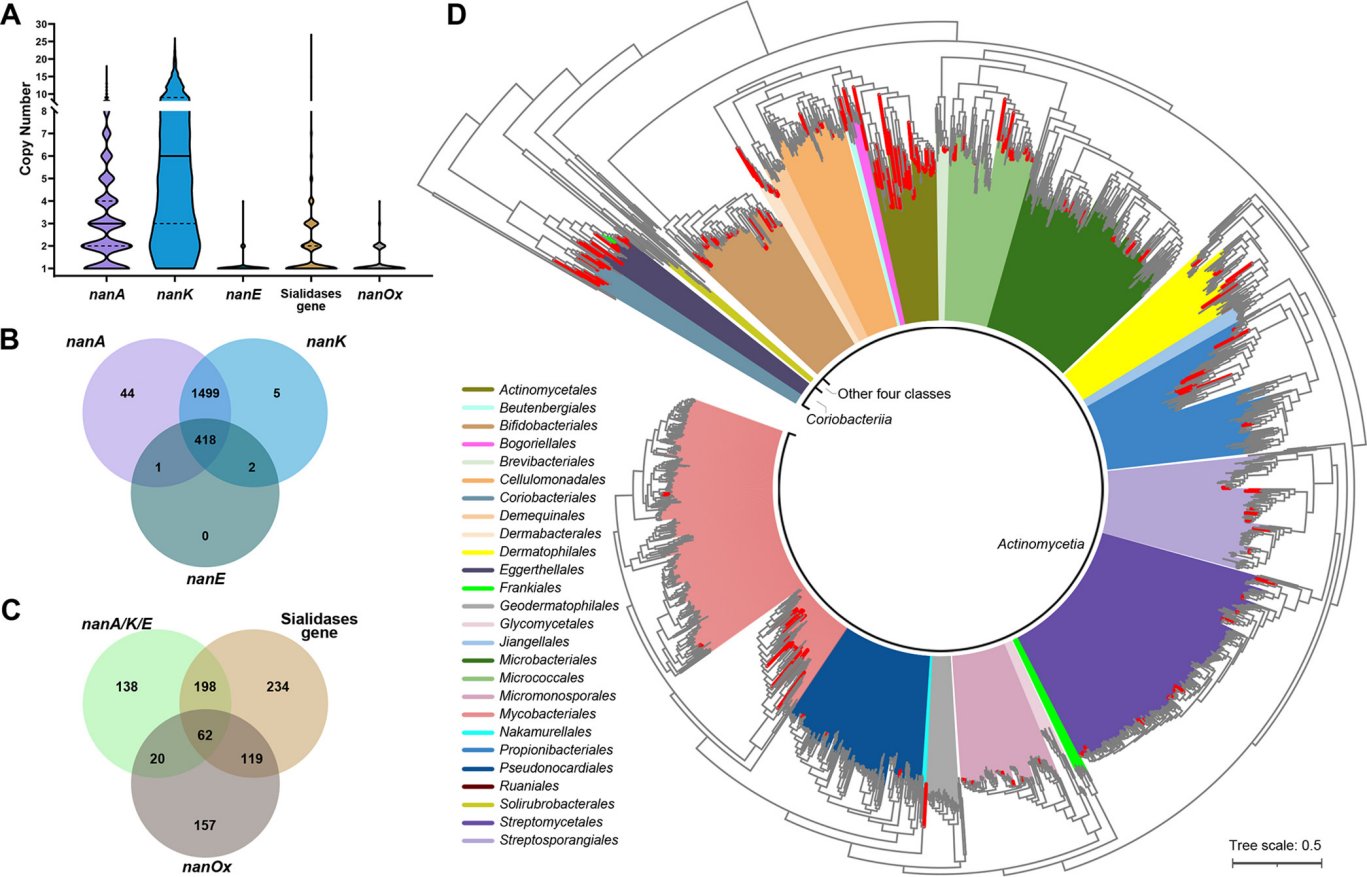
Distribution of putative sialic acid catabolism (SAC) genes and gene clusters among 1,969 actinobactieral species. (A) Violin plots showing the distribution of copy numbers of putative SAC genes within each genome. Only genomes containing the genes were considered. Center solid lines, median; dashed lines, quartile. (B and C) Venn diagram showing the presence of SAC genes and their distribution. *nanA/K/E*, containing all three genes (*nanA*, *nanK*, and *nanE*). (D) Phylogenetic reconstruction of the phylum *Actinobacteria* to map the taxonomic distribution of SAC. The tree was produced by using FastTree based on 138 universally conserved proteins of 1,969 type strains of *Actinobacteria*. Species with putative SAC are marked as red (canonical paradigm) and green (*Bacteroidetes* paradigm), and colored branches indicate different orders. The tree is rooted with nonactinobacterial strains (Vibrio cholerae N16961, Staphylococcus aureus USA300, and Escherichia coli MG1655). Scale bar indicates 50% sequence divergence.

We first examined the distribution of the three necessary genes for canonical SAC pathway, *nanA*, *nanK*, and *nanE* (hereafter *nanA/K/E*), in 7,180 high-quality and nonredundant available actinobacterial genomes (see Materials and Methods). We found that putative *nanA* and *nanK* genes were widely distributed in all six classes of *Actinobacteria* but putative *nanE* genes were distributed in only two classes, *Actinomycetia* and *Coriobacteriia*. To avoid statistical bias caused by intensive sequencing of single species, we used the 1,969 type strains (which represent species) in the data set to calculate the positive rate of *nan* genes in actinobacteria. The result showed that putative *nanA* and *nanK* occurred in nearly all of the actinobacterial species (99.64% and 97.71%, respectively) but putative *nanE* occurred in only 21.38% (421/1,969) of the species. In addition, the copy numbers of the three genes among the respective positive species also differed significantly (Fig. 1A), with that of *nanK* the highest and that of *nanE* the lowest. Nearly all (418/421) the *nanE*-positive species contained *nanA* and *nanK*, too (Fig. 1B). These results suggest that, in actinobacteria, *nanE* may represent the *nan* gene cluster better than *nanA* and *nanK*.

For the *Bacteroidetes* paradigm pathway, 88.17% of the species contained putative *rokA*, but 84.62% of the genes showed homology to *nanK*. Only seven species (all belonging to the class *Coriobacteriia*, six from *Eggerthellales*, and one from *Coriobacteriales*) contained *nanE-II*, with one copy per genome. Of these, only Rubneribacter badeniensis ResAG-85^T^ and “*Arabia massiliensis*” Marseille-P3078^T^ from the order *Eggerthellales* contained both *nanE-II* (clustered with *nanA*) and *rokA*, indicating that the alternative pathway is rare in *Actinobacteria*.

Furthermore, 31.13% (613/1,969) of the species contained putative sialidase genes with a median copy number of 1 per genome (Fig. 1A), and 5.06% (31/613) of these species encoded IT-sialidases. Around 37.80% (158/418) of the species that contained all *nanA/K/E* genes lacked sialidase genes (Fig. 1C). In addition, 18.18% (358/1,969) of the species contained putative *nanOx* responsible for converting 2,7-anhydro-Neu5Ac into Neu5Ac, among which 22.91% (82/358) of species contained all *nanA/K/E* (Fig. 1C). The above-mentioned two species with both *nanE-II* and *rokA* contained neither sialidase genes nor *nanOx*.

### Distribution of putative SAC in *Actinobacteria*.

To make the study tractable, we considered a strain to contain the SAC pathway only if its *nanA/K/E* genes are located within 10 open reading frames (ORFs) of each other (canonical paradigm) (23) or if it possesses clustered *nanA* and *nanE-II* (within 10 consecutive ORFs) and has *rokA*, too (*Bacteroidetes* paradigm). As shown in Table 1, putative SAC was distributed in 18.04% (1,295/7,180) of all actinobacterial strains tested and in 13.05% (257/1,969) of the species. Among them, only five strains and two species (*Rubneribacter badeniensis* and “*Arabia massiliensis*”) from the order *Eggerthellales* contained the putative *Bacteroidetes* pathway, and these strains/species did not have the canonical pathway. Among the 44 orders analyzed, 18 orders of the class *Actinomycetia* and two orders of the class *Coriobacteriia* were detected to contain SAC, with positive rates for species ranging from 1.78% to 69.23%. The positive rate was the highest in the order *Actinomycetales*, followed by *Dermabacterales* (66.67%), *Coriobacteriales* (52.63%), and *Eggerthellales* (50.00%), but was lower than one-third in the other orders (Table 1). The distribution of SAC was more different among families and genera (only those with at least three species in the final data set were considered), even among those belonging to the same order (Table S1). For example, the positive rate in the order *Mycobacteriales* was 9.32%, but the rates ranged from 0% (e.g., *Gordoniaceae*, *Dietziaceae*, *Tsukamurellaceae*) to 32.71% (*Corynebacteriaceae*) at the family level; although the positive rate in the order *Actinomycetales* approximated to 70%, no more than one-third of the species of the genera *Varibaculum* (33.33%) and *Arcanobacterium* (25.00%) of this order contained *nan* clusters. The 257 putative SAC-positive species belonged to 81 genera, interspersed among the *Actinobacteria* species phylogeny (Fig. 1D), and 36.19% (93/257) of these species were affiliated with four genera, *Actinomyces*, *Bifidobacterium*, *Corynebacterium*, and *Streptomyces* (Table S1). Collectively, these data suggest that the capacity for SAC in the phylum *Actinobacteria* is not innate. That is, the common ancestor of actinobacteria might not contain *nan*, but individual positive taxa likely acquired the cluster through horizontal gene transfer (HGT).

**TABLE 1:** Distribution of putative sialic acid catabolism among actinobacterial orders

Class	Order	No. of strains	No. of strains with SAC^*a*^	Positive rate^*b*^	No. of type strains	No. of type strains with SAC^*a*^	Positive rate in type strains^*b*^
*Acidimicrobiia*	*Acidimicrobiales*	3	0	ND	3	0	ND
	*Iamiales*	4	0	ND	4	0	ND
*Actinomycetia*	*Acidothermales*	1	0	ND	1	0	ND
	*Actinocatenisporales*	1	0	ND	1	0	ND
	*Actinomycetales*	229	179	78.17%	65	45	69.23%
	*Antricoccales*	4	0	ND	3	0	ND
	*Beutenbergiales*	7	1	14.29%	5	1	20.00%
	*Bifidobacteriales*	748	187	25.00%	91	24	26.37%
	*Bogoriellales*	13	1	7.69%	8	1	12.50%
	*Brevibacteriales*	70	33	47.14%	13	4	30.77%
	*Catenulisporales*	2	0	ND	2	0	ND
	*Cellulomonadales*	133	17	12.78%	61	5	8.20%
	*Cryptosporangiales*	3	0	ND	3	0	ND
	*Demequinales*	28	0	0.00%	17	0	0.00%
	*Dermabacterales*	34	21	61.76%	12	8	66.67%
	*Dermatophilales*	114	12	10.53%	60	8	13.33%
	*Frankiales*	30	0	0.00%	11	0	0.00%
	*Geodermatophilales*	72	0	0.00%	34	0	0.00%
	*Glycomycetales*	16	0	0.00%	14	0	0.00%
	*Jatrophihabitantales*	4	0	ND	1	0	ND
	*Jiangellales*	17	0	0.00%	13	0	0.00%
	*Kineosporiales*	13	0	0.00%	6	0	0.00%
	*Microbacteriales*	792	53	6.69%	173	12	6.94%
	*Micrococcales*	499	121	24.25%	81	19	23.46%
	*Micromonosporales*	270	33	12.22%	103	10	9.71%
	*Motilibacterales*	2	0	ND	2	0	ND
	*Mycobacteriales*	1,759	237	13.47%	429	40	9.32%
	*Nakamurellales*	7	1	14.29%	6	1	16.67%
	*Propionibacteriales*	309	20	6.47%	129	14	10.85%
	*Pseudonocardiales*	247	9	3.64%	169	3	1.78%
	*Ruaniales*	8	3	37.50%	2	1	ND
	*Sporichthyales*	1	0		1	0	ND
	*Streptomycetales*	1,286	184	14.31%	265	25	9.43%
	*Streptosporangiales*	195	19	9.74%	118	13	11.02%
*Coriobacteriia*	*Coriobacteriales*	154	116	75.32%	19	10	52.63%
	*Eggerthellales*	81	48	59.26%	26	13	50.00%
*Nitriliruptoria*	*Egibacterales*	1	0	ND	1	0	ND
	*Egicoccales*	1	0	ND	1	0	ND
	*Euzebyales*	2	0	ND	2	0	ND
	*Nitriliruptorales*	1	0	ND	1	0	ND
*Rubrobacteria*	*Gaiellales*	1	0	ND	1	0	ND
	*Rubrobacterales*	5	0	0.00%	3	0	ND
*Thermoleophilia*	*Solirubrobacterales*	12	0	0.00%	8	0	0.00%
	*Thermoleophilales*	1	0	ND	1	0	ND
							
Total		7,180	1,295	18.04%	1,969	257	13.05%

a SAC, sialic acid catabolism, here equal to cooccurrence and genetic linkage of *nanA/K/E* genes within 10 open reading frames (ORFs) (canonical paradigm) or to containing clustered *nanA* and *nanE-II* (within 10 consecutive ORFs) and having *rokA* as well (*Bacteroidetes* paradigm). The putative *Bacteroidetes* SAC is detected in only five strains (including two type strains) from the order *Eggerthellales*, and these strains do not contain the putative canonical SAC.

b The positive rate is calculated only when the total number of strains is at least 5. ND, no data.

### Distribution of putative SAC in animal-associated and non-animal-associated *Actinobacteria* species.

Based on the isolation source information of the 1,969 species used in this study, 1,923 (97.66%) species could be divided into two major source categories: animal host-associated (e.g., intestine, urinary tract, oral cavity, cow milk; 609 species) and non-animal-associated (e.g., soil, lakes, sea, plant-associated; 1,314 species). Among them, putative SAC was found in 24.79% (151/609) of the animal host-associated species but only in 7.84% (103/1,314) of the non-animal-associated species (*P < *2.2e−16, Fisher’s exact test). This is in accordance with the fact that sialic acids are generally found in glycoconjugates in animals. Unexpectedly, however, we did not detect *nan* in any member of *Mycobacterium* or *Nocardia*, two genera both containing a large number of animal host-associated species, although we analyzed 354 strains/74 species (193/62 host-associated) for *Mycobacterium* and 132 strains/76 species (65/39 host-associated) for *Nocardia* (Table S1). In addition, among the species with putative SAC, 40.08% (103/257) were non-animal-associated, including those of the genera *Streptomyces* (with a *nan*-positive rate of 8.37%), *Rhodococcus* (9.68%), *Micromonospora* (13.43%), and *Tessaracoccus* (66.67%) (Table S1). Notably, all *nan*-positive *Streptomyces* species (except for one of unknown source) were isolated from free-living habitats such as soil, although the positive rate (8.37%) was much lower than that of animal-associated genus *Actinomyces* (83.33%).

Moreover, 17.51% (45/257) of the putative SAC-positive species possessed *nanOx*, and 64.44% (29/45) of these species were non-animal-associated, indicating that a small number of actinobacteria, mainly free-living, have the capacity for anhydro-SAC. However, the 31 actinobacterial species with IT-sialidase genes were all animal associated, of which 24 species also contained *nan* and only Devriesea agamarum (order *Dermabacterales*) contained both *nan* and *nanOx* (outside the cluster). The information suggests that, in actinobacteria, the ability to release anhydro-sialic acids might be limited to a few animal-associated species, with very few species capable of both releasing and utilizing anhydro-sialic acids.

### Evolution of the *nan* gene clusters in *Actinobacteria*.

**FIG 2:**
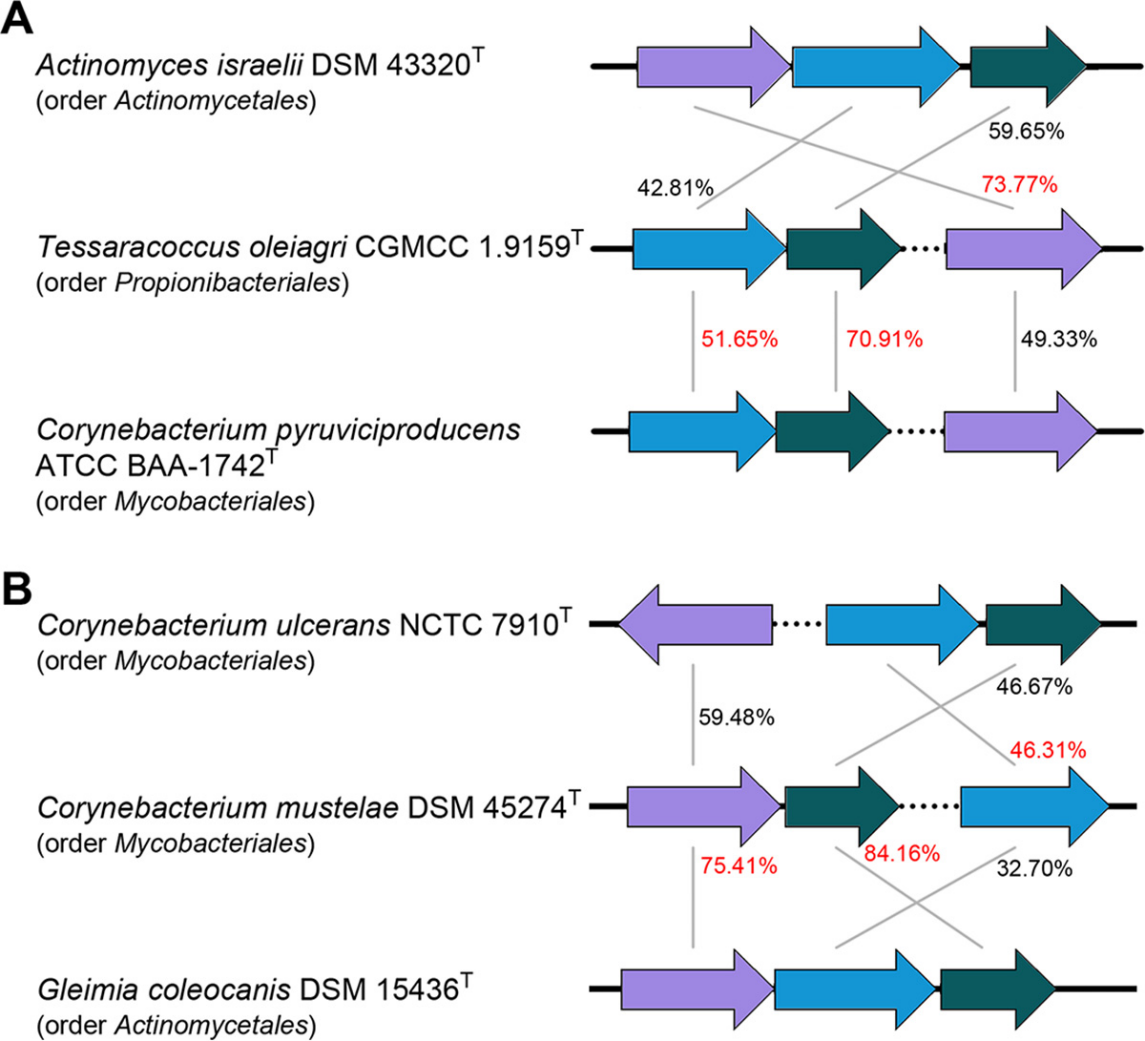
Comparisons of amino acid sequence identities of *nanA*, *nanK*, and *nanE* of Tessaracoccus oleiagri CGMCC 1.9159^T^ (A) and Corynebacterium mustelae DSM 45274^T^ (B) with the most similar counterparts of other actinobacteria. Sequence identities between homologues are indicated. The highest values between these two strains and other strains are marked in red. Only the *nanA* (purple), *nanK* (blue), and *nanE* (dark green) within the *nan* gene clusters are shown. Genes inserted are marked as dashed lines.

As mentioned above, the putative *Bacteroidetes* SAC pathway was found in only two actinobacterial species (and 5 strains); hence, we focused the following analyses on the canonical pathway. Phylogenetic analysis of individual *nanA/K/E* genes in actinobacteria (Fig. S1) showed that copies of these genes from *nan* clusters tend to form closely related branches rather than mix with the branches of the other copies. It is thus likely that the *nanA/K/E* genes within the clusters have undergone evolutionary processes different from those of their homologous genes scattered outside the clusters. Moreover, phylogenies of individual *nanA/K/E* within the gene clusters were incongruent with each other (Fig. S2). For instance, as shown in Fig. 2, Tessaracoccus oleiagri CGMCC 1.9159^T^ (order *Propionibacteriales*) contained *nanA* most similar to that of Actinomyces israelii DSM 43320^T^ (order *Actinomycetales*) but *nanK* and *nanE* most similar to those of Corynebacterium pyruviciproducens ATCC BAA_1742^T^ (order *Mycobacteriales*), and Corynebacterium mustelae DSM 45274^T^ contained *nanA* and *nanE* most similar to those of *Gleimia coleocanis* DSM 15436^T^ (order *Actinomycetales*) and *nanK* most similar to that of Corynebacterium ulcerans NCTC 7910^T^. These observations suggest that the clustered *nanA/K/E* genes may also have evolutionary histories different from each other.

**FIG 3:**
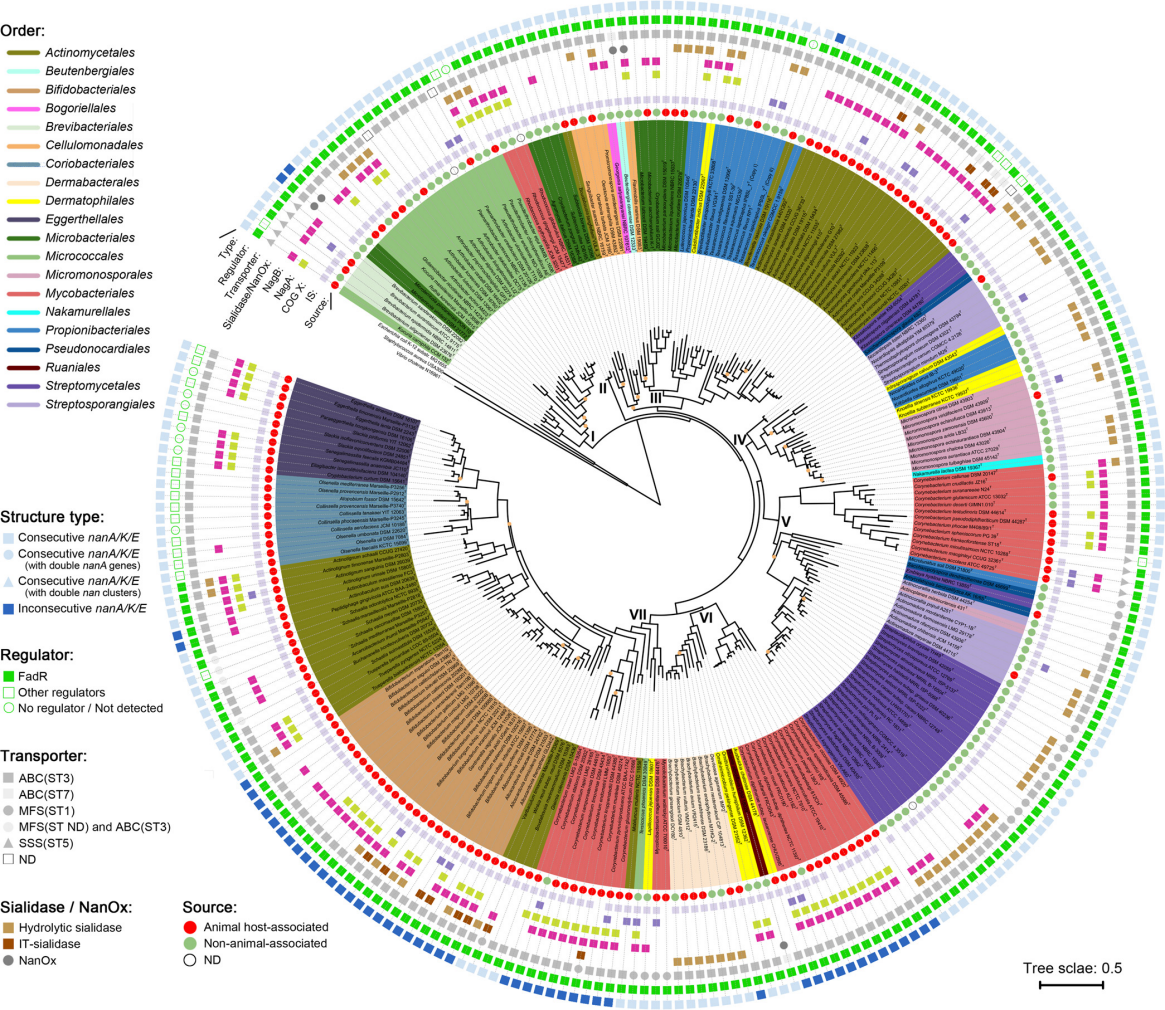
Phylogeny of the concatenated NanA-K-E proteins of 255 actinobacterial species, with features of each *nan* gene cluster and the source of each species indicated. The tree was produced using FastTree. Nodes with bootstrap values less than 60% are marked with orange circles. Colored background of strain names indicates different orders. “I” to “VII” indicate genetic clades. Scale bar indicates 50% sequence divergence. Circles from the outside to the inside indicate the type of structures of the *nan* clusters, the type of regulator genes (COG K), the family of transporters, the presence or absence of sialidase gene and *nanOx*, *nagB*, *nagA*, mobilome (COG X), and insertion sequences (IS) around/within the *nan* clusters, and the source of the strains, respectively. ABC, ABC transporter; SSS, sodium solute symporter; MFS, major facilitator superfamily; ST, sialic acid transporter; ND, not determined.

We next performed phylogenetic analysis based on the concatenated proteins encoded by the clustered *nanA/K/E*. The resulting trees (Fig. 3 and Fig. S3) showed apparent inconsistencies with the corresponding species trees that showed a clear separation of different actinobacterial orders (Fig. S4 and Fig. 1D). The NanA-K-E tree of 255 species could be divided into seven well-separated clades, clades I to VII (Fig. 3 and Fig. S4). The orders *Eggerthellales* and *Coriobacteriales* of the class *Coriobacteriia* and the order *Bifidobacteriales* were located in the terminal branches (clade VII) of the NanA-K-E tree, neighbored with some species of the order *Actinomycetales*. But the former three orders were located at the base of the species tree, distinct from the other orders (Fig. S4). Furthermore, in the NanA-K-E tree, taxa from the same order were often distributed in different clades, and the genus *Corynebacterium* was distributed in clades V to VII (Fig. 3). These phenomena indicate that some *nan* clusters of the same orders, and even of the same genera, might have different origins or experience different evolutionary processes. Meanwhile, the phylogeny showed clear signals for interorder HGT of *nan*: species Calidifontibacter indicus of the order *Dermatophilales* was stably located in a branch of four genera from the order *Propionibacteriales* in clade III, and similar situations were found for species Actinoplanes missouriensis of the order *Micromonosporales*, Tessaracoccus oleiagri of the order *Propionibacteriales*, and Ruania albidiflava of the order *Ruaniales* (Fig. 3). Nevertheless, in general, most strains from the same orders tended to cluster together in the NanA-K-E phylogeny, although they are often clustered in different clades. In addition, some shallow branches in the NanA-K-E tree and species tree had the same topological structures, such as a branch in clade VII formed by one *Peptidiphaga* species, two *Actinobaculum* species, and four *Actinotignum* species from the order *Actinomycetales* (Fig. S4). All these observations indicate that the spreading of *nan* among actinobacterial orders is due mainly to occasional HGT events of *nan* genes, which are further propagated in respective orders by subsequent intraorder HGT as well as vertical inheritance among closely related species.

Furthermore, we found a rough correlation between the phylogenetic clades of *nan* and the source of the species. Clades VI and VII contained mainly (96/106, 90.57%) animal-associated species, while clades IV and V contained mainly (58/72, 80.56%) non-animal-associated species (with eight animal-associated species forming a monophyletic branch) (Fig. 3). Meanwhile, the isolation sources of *nan*-positive species in clades I and III (except for the species of *Actinomyces* in a subclade) showed frequent alternation between animal- and non-animal-associated environments, suggesting a high frequency of *nan* gene exchange between the host-associated and free-living species.

### Gain and loss events of the *nan* clusters in *Actinobacteria*.

**FIG 4:**
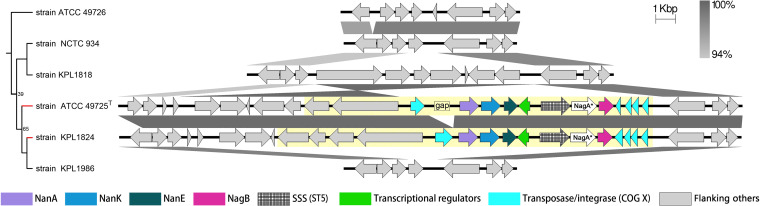
Genetic organization of the regions containing *nan* clusters indicating HGT events in Corynebacterium accolens strains. The figure was obtained by EasyFig, where grayscale bars represent regions of shared similarity according to BLASTN. Genomic islands detected by Islandviewer are marked with yellow background. The tree on the left shows the phylogenetic relationships of the corresponding strains based on 138 universally conserved proteins of *Actinobacteria*, and strains with the *nan* cluster are marked as red. Branch lengths are ignored, and bootstrap values less than 70% are shown. Putative *nagA* genes annotated with no observed Pfam domains are indicated with asterisks. SSS, sodium solute symporter transporter; ST, sialic acid transporter.

To further decipher the evolutionary history of the *nan* gene cluster in *Actinobacteria*, we assessed the gain and loss events of this cluster among the species using COUNT (33). As a result, 135 gains and 18 losses were detected, which were scattered among the species tree with 88 gains and 14 losses occurring at the species level (Fig. S5). No gene gain or loss events were inferred to have occurred in the last common ancestor of any orders, supporting that *nan* clusters were acquired by *Actinobacteria* strains through HGT long after the early divergence of the phylum. Furthermore, 95.29% (243/255) and 9.80% (25/255) of the *nan*-positive species embraced insertion sequences (ISs) and mobilome genes (Clusters of Orthologous Group [COG] X) around *nan*, respectively (Fig. 3), providing more evidence for potential HGT events of *nan* (34, 35). For instance, in a species tree branch containing six strains of Corynebacterium accolens, only two strains contained *nan* clusters, adjacent to which were a series of genes encoding transposases/integrases (all annotated as IS and proteins of COG X), and the genes at both ends of the regions were quite conserved (Fig. 4). Moreover, these genomic regions of *nan* were identified as genomic islands by Islandviewer (36), underpinning that SAC was introduced into the two *Corynebacterium* strains by HGT.

**FIG 5:**
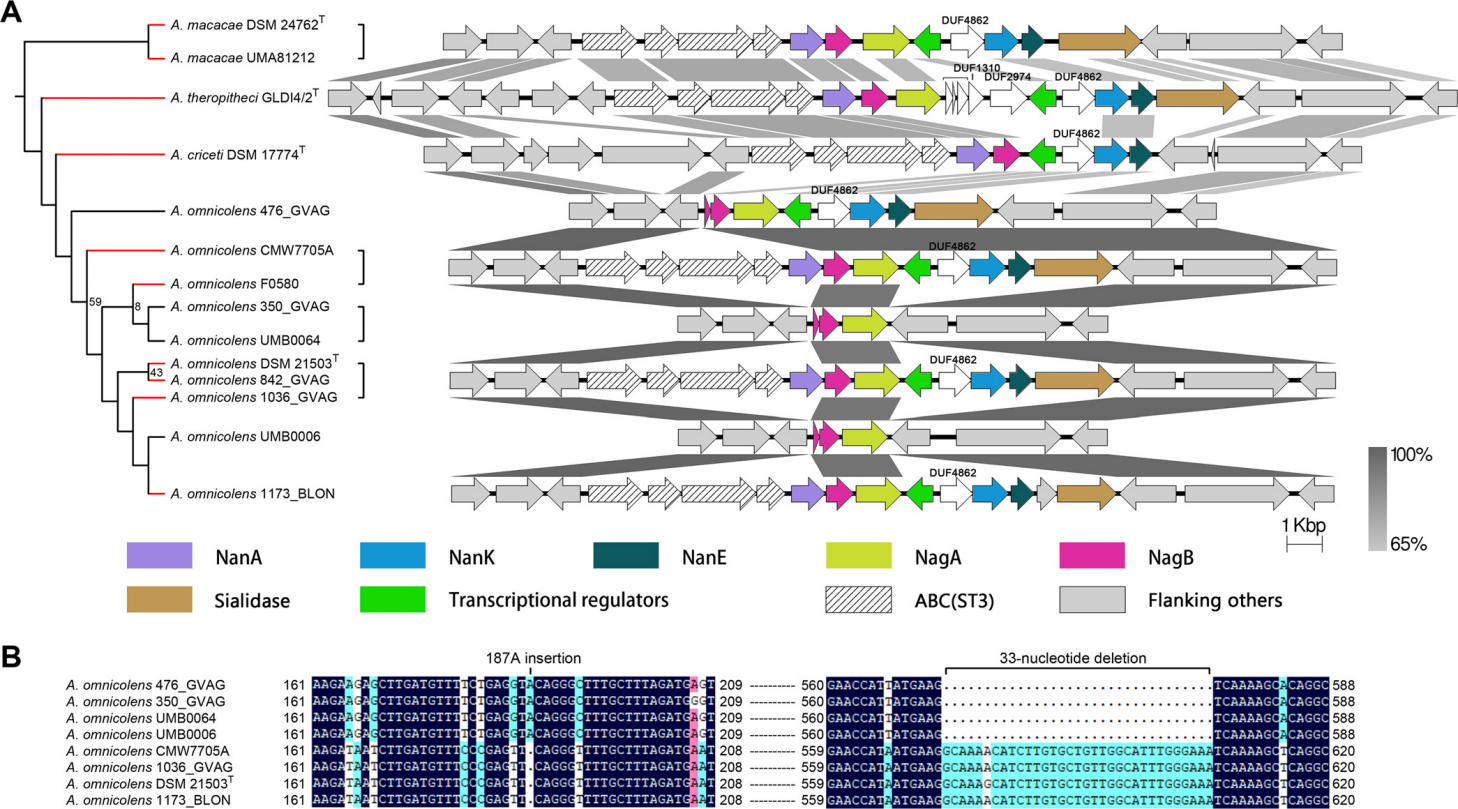
Gene loss events occurring in the *nan* clusters of the genus *Alloscardovia*. (A) Comparison of the genetic organization of the regions containing *nan* genes. The figure was obtained by EasyFig, where grayscale bars represent regions of shared similarity according to BLASTN. Pfam annotations for the other genes within the *nan* clusters are also given, and genes with no Pfam annotations are indicated with a bracket. The tree on the left shows the phylogenetic relationships of the corresponding strains based on 138 universally conserved proteins of *Actinobacteria*, and strains with *nan* clusters are marked as red. Square brackets indicate that structures of the regions of the bracketed strains are the same. Branch lengths are ignored, and bootstrap values less than 70% are shown. ABC, ABC transporter; ST, sialic acid transporter. (B) Sequence alignment of the *nagB*. Gaps are indicated by dots. Identical (100%), conserved (75 to 99%), and blocks (50 to 74%) of similar amino acid residues are shaded in dark blue, pink, and cyan, respectively. Numbers show the positions of the bases in the gene alignment.

Meanwhile, several gene loss events were detected in the genus *Alloscardovia* (Fig. 5). All analyzed *Alloscardovia* species contained complete *nan* clusters, except for 4 out of the 10 strains of Alloscardovia omnicolens, a species dwelling everywhere in the human body (37). Comparison of the genomic regions of the *nan* clusters illustrated that the *nan-*negative strain 476_GVAG lost *nanA* and transporter genes, and the other three *nan-*negative strains lost *nanA/K/E* and transporter genes (Fig. 5A). In addition, in the remaining *nagB* of the negative strains, there was an 187A insertion that resulted in a premature termination codon mutation and a 33-nucleotide deletion at positions 573 to 606 (Fig. 5B). These two mutations probably inactivate *nagB*, supporting that these strains are gradually losing the whole *nan* cluster, although there was another copy of *nagB* (with amino acid identity of <39.5%) in their genomes.

### Compositional and structural diversity of the *nan* clusters.

**FIG 6:**
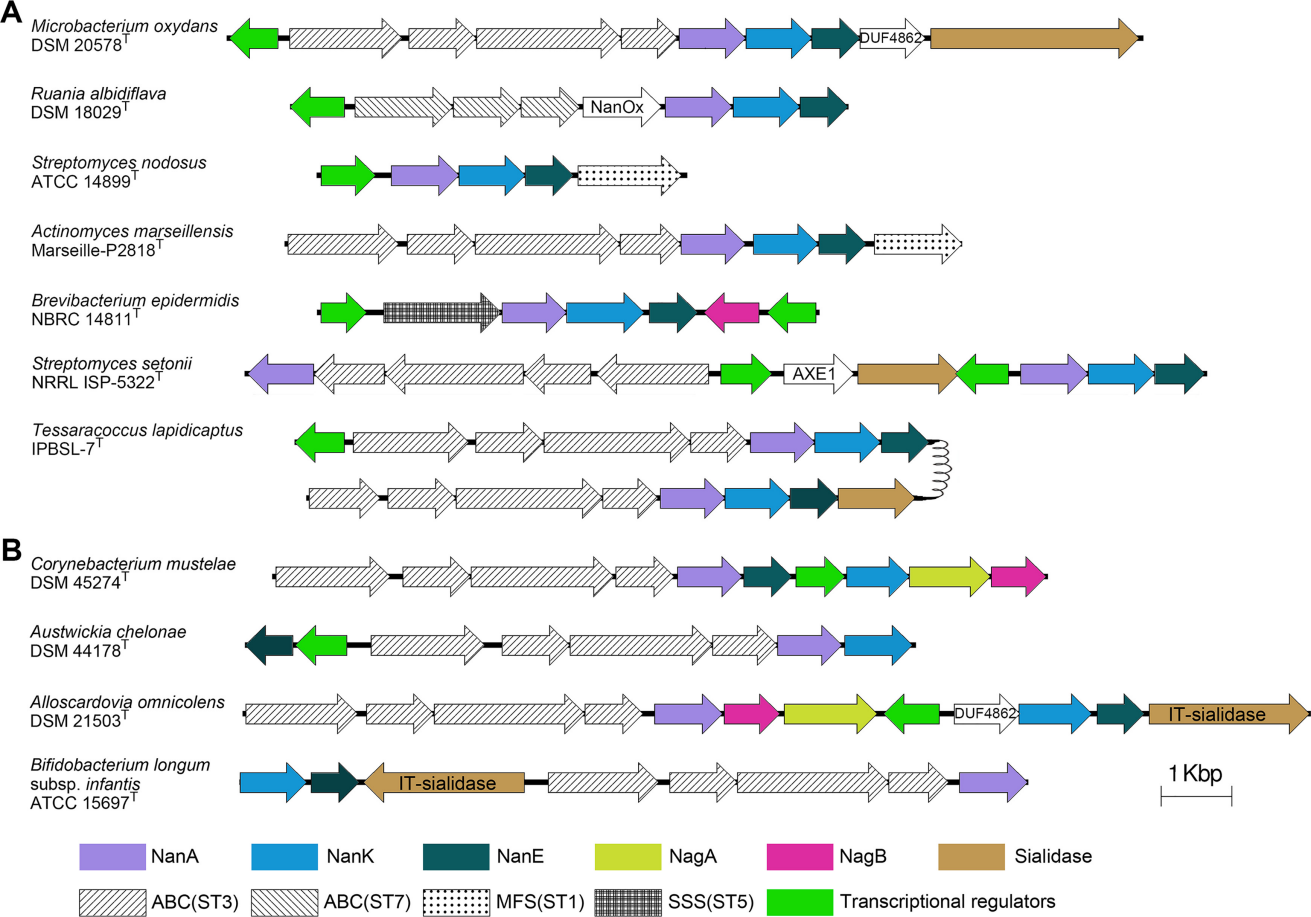
Compositional and structural diversity of the *nan* clusters in actinobacteria. (A) Consecutive *nanA/K/E* type: *nan* clusters with *nanA/K/E* located next to each other in sequence. (B) Inconsecutive *nanA/K/E* type: *nan* clusters with *nanA/K/E* separated by other genes. ABC, ABC transporter; SSS, sodium solute symporter; MFS, major facilitator superfamily transporter; ST, sialic acid transporter.

We found a high diversity of *nan* by comparing the genetic organization of the gene clusters among actinobacterial species. The clusters contained genes encoding three of the four major superfamilies of bacterial transporters (ABC, MFS, and SSS; Fig. 3 and 6). The ABC superfamily was widely distributed in 88.24% of actinobacterial SAC systems, containing two different ST families. Most of the ABC transporters were featured as Pfam SBP_bac_5 (PF00496, ST3) that transports Neu5Ac, and the remaining were SBP_bac_1 (PF01547, ST7) that specializes in the uptake of 2,7-anhydro-Neu5Ac, occurring in six species of the genera *Beutenbergia* (*n* = 1), *Ruania* (*n* = 1), and *Actinomyces* (*n* = 4). The MFS superfamily was present in 22 species of clades V and VII, including 18 species with ST1 family transporters and four *Actinomycetales* species that embraced both ABC (ST3) and MFS transporters (ST family not determined). The SSS superfamily (ST5) occurred in nine species that formed two monophyletic branches in clades I and V of the *nan* phylogeny. Three *nan* clusters were found to contain no transporter genes. Two of the clusters were located only at contig boundaries of the genome sequences, where genes might be missing. But the transporter system of Arthrobacter castelli DSM 16402^T^ was truncated, leaving two genes of PF00528 (BPD_transp_1), possibly implying gene loss from the original cluster or HGT of partial transporter genes into the cluster.

Among the *nan* clusters of 255 actinobacterial species, 71 and 123 *nan* clusters contained *nagA* and *nagB*, respectively, 68 of which contained both (Fig. 3). Eighty-two *nan* clusters contained sialidase genes, of which 12 clusters (all from animal host-associated species) contained IT-sialidase genes (Fig. 3). Only five *nan* clusters harbored *nanOx*, and all of them occurred in free-living species, of which Beutenbergia cavernae and Ruania albidiflava also possessed ST7 transporters for anhydro-Neu5Ac (Fig. 3). Regarding the regulation of SAC in actinobacteria, nearly all *nan* clusters (96.58%, 226/234) in the class *Actinomycetia* contained the regulator gene *fadR* (COG2186), whereas those in the class *Coriobacteriia* contained *glpR* (COG1349), *whiA* (COG1481), *acrR* (COG2207), etc. instead or did not contain regulator genes (Fig. 3). These results suggest that the related components of *nan* clusters in actinobacteria have evolved multiple times, further enhancing the diversity of the clusters.

Moreover, according to the arrangement of *nanA/K/E* genes, structures of the *nan* clusters in actinobacteria could be grouped into two major types, consecutive *nanA/K/E* and inconsecutive *nanA/K/E* (Fig. 6). In general, the *nanA/K/E* genes were located next to each other in sequence (Fig. 6A). Among the species with this *nan* type, Glutamicibacter nicotianae (order *Micrococcales*, clade I) and seven *Streptomyces* species in a clade V branch possessed an additional copy of *nanA* that was located next to the *nan* clusters (Fig. 3). This *nanA* copy of *Streptomyces* species shared 55.04 to 57.04% (mean, 55.89%) amino acid identity with those in the adjacent *nan* clusters but a higher mean identity of 63.01% with those of three *Kitasatospora* species in clade IV. It is thus suggested that HGT of *nanA* has occurred between *Streptomyces* and *Kitasatospora*, two genera previously reported to have undergone extensive recombination (38, 39). In addition, almost all *nan*-positive actinobacteria contained only one *nan* cluster, but Tessaracoccus lapidicaptus IPBSL-7^T^ contained two copies of the cluster. These two *nan* copies were located in different contigs of the genome sequence and shared 81.71% amino acid identity of NanA-K-E. Interestingly, one of the copies shared 99.10% NanA-K-E identity to the *nan* from Tessaracoccus flavus RP1^T^ (Fig. S6), while the two type strains had a genome-wide average amino acid identity of only 75.35%. A similar phenomenon was also seen in T. lapidicaptus strain T2.5-30. This result implies HGT of *nan* between different *Tessaracoccus* species rather than duplication of *nan* within the genome.

In 53 (20.78%) *nan*-positive species, *nanA/K/E* genes were separated by *nagA/B*, sialidase genes, the transporter genes, the regulator genes, and/or other genes, forming *nan* clusters of the inconsecutive *nanA/K/E* type (Fig. 6B). Almost all (50/53) of these clusters were distributed in clades VI and VII, and most of them (47/53) were from three monophyletic branches comprising the genera *Bifidobacterium*, *Gardnerella*, *Alloscardovia*, and *Corynebacterium* (Fig. 3). These “mosaic” *nan* clusters showed flexible organization, suggesting multiple evolutionary origins of the SAC-associated enzymes.

## DISCUSSION

### Relationship between SAC and habitat.

Microbial SAC has long been regarded as a virulence determinant in a range of infectious diseases (2). This is accordant with our result that the positive rate of SAC is the highest in the order *Actinomycetales*, as the majority of *Actinomycetales* strains are disease associated (40–43). Similar instances also occur in some genera of the order *Bifidobacteriales*. For example, the genera *Alloscardovia* and *Gardnerella* show high positive rates of SAC (Table S1), and correspondingly, strains of them have been reported to cause bacteremia (44) and bacterial vaginosis (45, 46), respectively. However, our study also reveals lack of SAC in a number of notorious pathogenic actinobacteria, such as the tuberculosis-causing mycobacteria (47), nocardiosis-causing *Nocardia* spp. (48), and actinomycetoma-causing *Nocardia* spp., *Actinomadura* spp., and Streptomyces somaliensis (49). On the other hand, we provide further evidence that SAC also exists in some probiotics, which may provide nutritional immunity and competition for pathogens in the gut environment (29). It has been reported that sialic acid consumption could be used as a treatment of bacterial infection. For instance, the use of probiotics with the ability of SAC to outcompete the pathogens for sialic acids can effectively limit or reverse Clostridium difficile infection (50). Therefore, our study reinforced the acknowledgment in a systematic way that the pathogenic property of microbial SAC should not be generalized to host-associated microbes, and case-by-case analysis is needed for different taxa.

Against the accepted knowledge that SAC is near exclusive to host-associated bacteria (11, 23), the phylum-level investigation here systematically confirms that SAC also exists in many free-living bacterial species. SAC has previously been found in soil actino bacterium Corynebacterium glutamicum (30), and our recent studies also showed that, in *Streptomyces* species, the *nan* cluster was significantly associated with free-living strains (31, 32). Frequent *nan* gene exchange between host-associated and free-living species, as observed in the orders *Brevibacteriales* and *Micrococcales* (Fig. 3), may have occurred in the early evolution of SAC in actinobacteria. Still, the *nan* phylogeny correlates to some extent with the lifestyles of actinobacteria, suggesting that different environmental pressures may confer relatively independent evolution/dispersal histories on the *nan* clusters. The presence of IT-sialidase and NanOx genes within *nan* clusters correlates with host-associated and free-living species, respectively, supporting different evolutionary processes of *nan* in different habitats. The occurrence of both NanOx and characterized anhydro-Neu5Ac transporter (ST7) genes within the *nan* clusters of free-living species Beutenbergia cavernae and Ruania albidiflava strongly suggests their capacity for uptake and dissimilation of anhydro-sialic acids. These findings probably mean that some free-living actinobacteria can use (anhydro-)sialic acids, perhaps from surrounding fungi or decomposing animals (30), efficiently as nutrient sources to survive adverse conditions and thus play an important role in the spread of SAC. Yet, although the *nan* clusters identified here show adequate integrity, most of them still need to be verified experimentally for their true function in SAC or even in catabolism of other nonulonosinic acids.

### HGT-mediated evolution of SAC in *Actinobacteria*.

It has been reported that the biosynthesis of sialic acids occurred roughly 500 million years ago, prior to the divergence of *Coelomata* (protostomes and deuterostomes) (2, 51, 52). By that time, the main orders of *Actinobacteria* had already diverged from each other (53). Therefore, the *nan* clusters responsible for SAC in actinobacteria should be considered “foreign goods.” Combined with the limited distribution of the clusters in actinobacteria (∼13%) and the distinct topologies between the NanA-K-E and species phylogenies, it is reasonable to deduce that HGT plays an important role in the evolution and spread of *nan* in actinobacteria. A *nan* cluster may transfer in entirety (e.g., Corynebacterium accolens) (Fig. 4), while its gene elements could further exchange between species. Meanwhile, an intact cluster may be combined by genes (including not only the *nan* genes but also genes of transporters, regulators, and Nag enzymes) that were acquired by multiple HGT events from different species at different evolutionary periods. Both of these two ways may lead to the formation of genomic islands as well as mosaic gene clusters in chromosomes. It has been argued that the most prevalent transporters (ST3) in *Actinobacteria* are autochthonous to this phylum (18); hence, the complete *nan* clusters containing ST3 transporter genes might have arisen in *Actinobacteria* via the latter way. Accompanied with different gene combinations and gene losses or modifications, the *nan* clusters have developed a high compositional and structural diversity, which may further alter its functional efficiency in different actinobacterial species. Until now, there was no clear evidence for the origin of SAC in bacteria. The multiple-clade topology of the NanA-K-E phylogeny may suggest either different evolutionary directions of *nan* after it entered into actinobacteria or multiple *nan* “invasion” events from other organisms.

The evolutionary pattern of SAC in actinobacteria probably represents those of a series of noncore pathways in bacteria. Through occasional HGT, a species can acquire specific adaptive genes from a distantly related organism (54–56). After that, the acquired genes can spread between generations or closely related species by vertical inheritance, accompanied with gene loss. These genes can also spread between higher taxa (such as families and orders) by further HGT, forming a new metabolic pathway or altering an original pathway in the recipient species to improve its environmental adaptability. During this process, the adaptive genes may spread to other habitats, where they may perform other adaptive functions or remain in the genome as a “strategic reserve” of species. Due to the different occurrence times of HGT and the different habitats and taxonomic status of the recipients, the positive rates of the genes or clusters among different taxa may vary greatly. Such evolutionary pattern has played important roles in generating tremendous genetic, ecological, and metabolic diversity in bacteria, especially in *Actinobacteria* (57, 58).

### Diversity of the *nan* clusters in actinobacteria.

Our results demonstrate again that, in addition to the necessary genes *nanA/K/E*, an intact *nan* cluster probably consists of one or more transporter genes, in most cases a regulator gene, sometimes *nagA/B* and/or sialidase genes, and occasionally *nanOx* (2, 9, 11, 19). In line with a very recent report (18), we detected ST1 (of MFS), ST5 (of SSS), and ST7 (of ABC) transporter families in addition to the widespread ST3 (of ABC) transporters in *Actinobacteria*. The MFS transporters of undetermined ST family in four *Actinomycetales* species may imply more ST diversity that remains to be studied. The different regulatory genes in the *nan* clusters may suggest different regulatory mechanisms of SAC between the classes *Actinomycetia* and *Coriobacteriia* or even among *Coriobacteriia* taxa, although the latter were all derived from animal hosts (https://www.ezbiocloud.net/). In addition, our recent work on *Streptomyces* also illustrates that actinobacterial *nan* clusters may contain accessory genes such as *nanS* (encoding acetyl-transferase/hydrolase) (31, 32). Different combinations and orders of all these genes expand the diversity of the *nan* clusters, attesting to a complex evolutionary history of *nan* in *Actinobacteria*. However, it should be mentioned that this *in silico* analysis is limited to the genomic level. Further experiments are needed to verify the functions of the diverse *nan* clusters, especially whether the additional genes, such as *nanA*, *nagA/B*, *nanOx*, and sialidase genes, in the clusters could enhance the efficiency of (anhydro-)SAC and environmental adaptation in the actinobacteria.

## MATERIALS AND METHODS

### Collection of actinobacterial genome sequences.

Genome information of strains (17,954 in total) belonging to the phylum *Actinobacteria* was obtained from EzBioCloud’s public genome database (March 2021) (https://www.ezbiocloud.net/), which includes only taxonomically corrected and quality-controlled genomes (59). Taxonomic positions and source information of these strains were also obtained from the database. Corresponding contigs or scaffolds of the genome sequences were acquired from the NCBI (with ncbi-genome-download tool, https://github.com/kblin/ncbi-genome-download/) and JGI public databases.

We next applied stringent quality control measures to ensure a high-quality and minimally biased set of genomes. CheckM (60) was first used to assess genome completeness and contamination. Only genomes that were more than 95% complete and had less than 5% contamination were retained. The GToTree package v1.5.47 (61) was then used to filter out genomes containing less than 75% of the 138 actinobacterial marker genes included in the package. Then, for each genus, genomes of all type strains were retained, and genomes of non-type strains were dereplicated using dRep (62) with a threshold of 99.5% pairwise genome-wide average nucleotide identity (ANI) value calculated by fastANI (63). Finally, genomes that showed discrepancy between their given taxonomy and their actual phylogenetic placement in the actinobacterial genome tree were also filtered out. A final set of 7,180 nonredundant actinobacterial genomes were obtained, of which 1,969 were from type strains that were assigned to 6 classes, 44 orders, 73 families, and 337 genera (Table S1). Three nonactinobacterial strains (Vibrio cholerae N16961, Staphylococcus aureus USA300, and Escherichia coli MG1655) were used as outgroups.

### Genome annotation and identification of putative sialic acid catabolic genes.

In an attempt to overcome the bias from different annotations, all the protein coding sequences (CDSs) were repredicted and reannotated using Prokka (64). In order to identify putative SAC genes (including *nanA*, *nanK*, *nanE*, *nagA*, *nagB*, and sialidase genes), all CDSs were subjected to PfamScan (version 31.0) (65) with an E value of <1e−10 against the PFAM-A database of protein families to identify those containing conserved protein domains of SAC (Table S2). The resulting protein sequences were further analyzed using reverse-position-specific BLAST with an E value of <1e−10 against NCBI’s Conserved Domain Database (CDD) (66) to confirm the presence of SAC-associated domains (Table S2). We followed the method proposed by Tailford et al. to distinguish IT-sialidases from hydrolytic sialidases (16). Briefly, all proteins from the CAZy GH33 family were downloaded and filtered by CD-HIT (67) with default settings to remove highly similar sequences. Then, after alignment, segments of *Rg*NanH-type domain were extracted to build a model for further HMM searching against the putative sialidase genes identified above by Pfam and conserved domains. Finally, the resulting sequences were manually checked for the presence of the segmented *Rg*NanH-type domain. Genes of NanE-II, RokA, and NanOx were determined by DIAMOND BLASTP (E value of <1e−5 and identity of >30%) using typical protein sequences listed in Table S2 as queries (19, 21, 22). If the *nanA/K/E* genes in a genome were within 10 ORFs of each other (canonical paradigm), or alternatively, if a genome contained *nanA* and *nanE-II* within 10 consecutive ORFs and contained *rokA* as well (*Bacteroidetes* paradigm), we determined that the strain had the ability to catabolize sialic acids (23). The nearest transporter genes around *nanA/K/E* genes were determined to encode sialic acid transporters, and the ST families were determined by Pfam searching according to Severi et al. (18). Regulatory genes within and/or around the cluster (within three ORFs) were annotated using COG annotation (COG K). Insertion sequences (IS) and transposable elements were detected and annotated using ISFinder (68) with an E value of <1e−5 and using COG annotation (COG X), respectively. The COG annotation was performed by sequence comparison using DIAMOND BLASTP (E value of <1e−5, coverage >50%, and identity >50%) against the recently updated COG database (69). The presence of genomic island was predicted using the IslandViewer online tool (36). Linear comparison of corresponding genomic regions was performed using BLASTN and visualized by the EasyFig software (70). Visualization of sequence alignment was produced by DNAMAN version 9.0 (Lynnon Biosoft, Canada).

### Phylogenetic analysis.

In order to infer the evolutionary relationships of actinobacterial species, the GToTree package v1.5.47 (61) was used with default parameters. All CDSs were searched against the actinobacterial single-copy gene set in GToTree that contains 138 marker genes using HMMER3 (71). A concatenated protein alignment from the marker genes was constructed using Muscle (72) and subsequently trimmed using TrimAl to remove poorly aligned regions (73). The approximately maximum-likelihood phylogenomic tree was built using FastTree (74). The average amino acid identity between genomes was calculated using CompareM (https://github.com/dparks1134/CompareM).

Multiple protein sequence alignments of each *nanA/K/E* gene were performed using MAFFT l-INS-I (75) and trimmed with TrimAl (73). Phylogenetic reconstruction was carried out using FastTree (74). For the *nan* phylogeny, only the *nanA/K/E* genes clustered within 10 ORFs of each other (23) were considered, and the phylogeny was determined based on the concatenated NanA-K-E sequences. All trees were further rendered by the iTOL (Interactive Tree Of Life) online tool (http://itol.embl.de/itol.cgi) (76) and with the ggtree package (77). To elucidate the evolutionary process of the *nan* cluster, we performed ancestral reconstructions using the COUNT software (33) with Wagner parsimony. An absence/presence (0/1) matrix of the *nan* cluster and the species tree of type strains were used as input files.

## Supplementary Material

Reviewer comments
