# Supplementary material for: Distribution and Evolutionary History of Sialic Acid Catabolism in the Phylum Actinobacteria
Source: Microbiol Spectr. 2022 Jan 12;10(1):e02380-21. doi: 10.1128/spectrum.02380-21 (PMC8754123; doi:10.1128/spectrum.02380-21)
Supplement: SUPPLEMENTAL FILE 3 — Supplemental material. Download SPECTRUM02380-21_Supp_3_seq13.pdf, PDF file, 4.7 MB [file spectrum02380-21_supp_3_seq13.pdf]

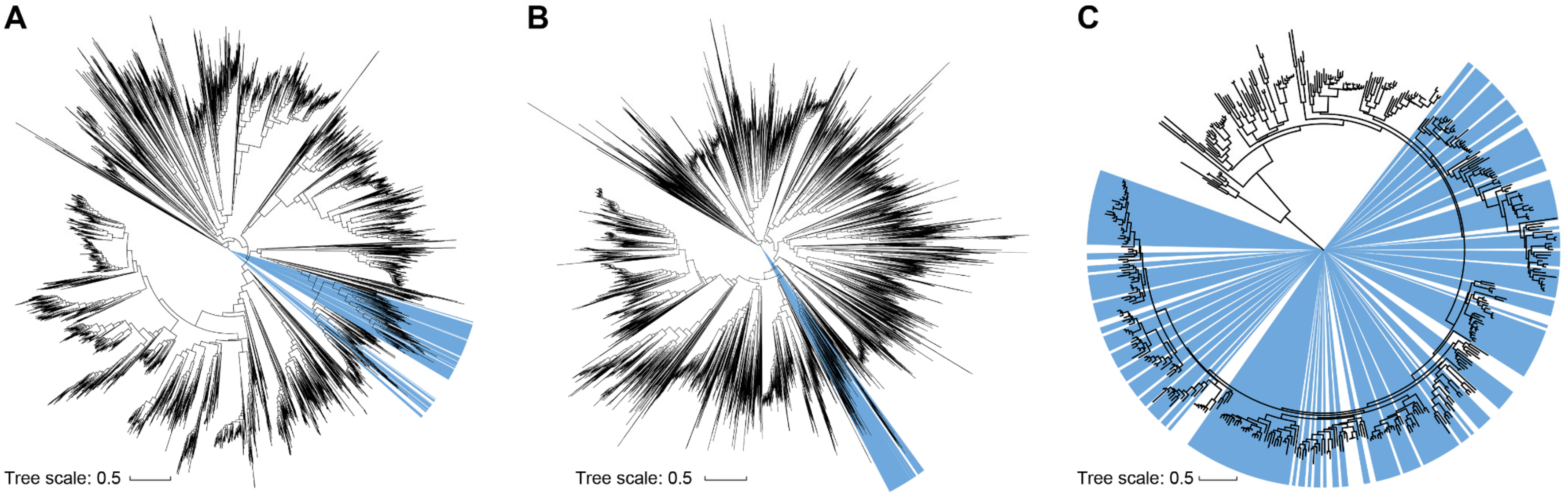


**Figure S1. Phylogenies of the NanA (A), NanK (B), and NanE (C) proteins of actinobacterial species.** Blue branches indicate proteins encoded by genes within the *nan* clusters. Scale bar indicates 50% sequence divergence.

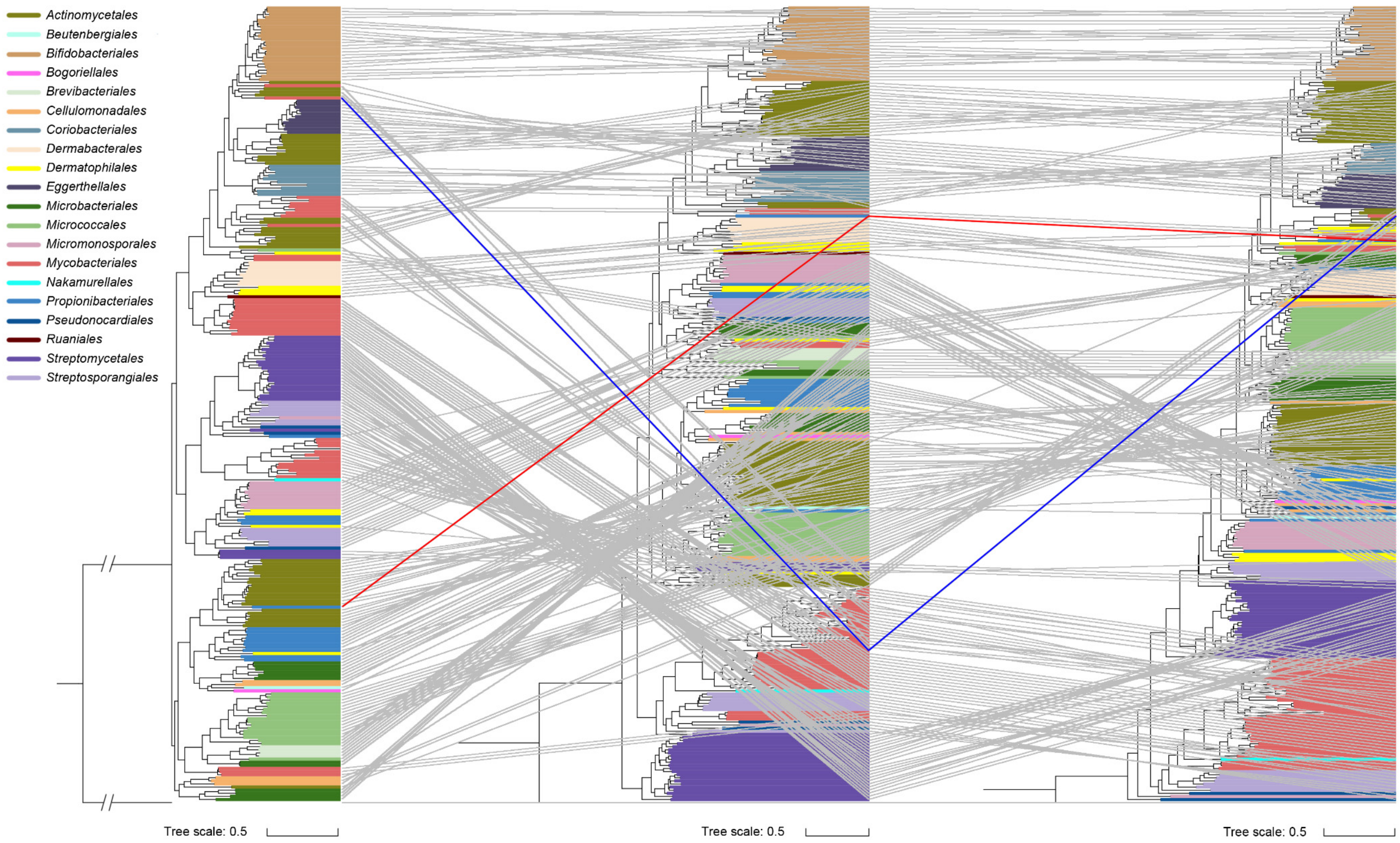


**Figure S2. Comparison of the phylogenies of NanA (left), NanK (middle), and NanE (right) encoded by genes within the *nan* clusters of 255 actinobacterial species.** Gray lines show the comparisons of the positions of strains on the gene trees (red, *Tessaracoccus oleiagri* CGMCC 1.9159$^{T}$; blue, *Corynebacterium mustelae* DSM 45274$^{T}$). Different colors in the trees indicate different orders. Scale bars indicate 50% sequence divergence.

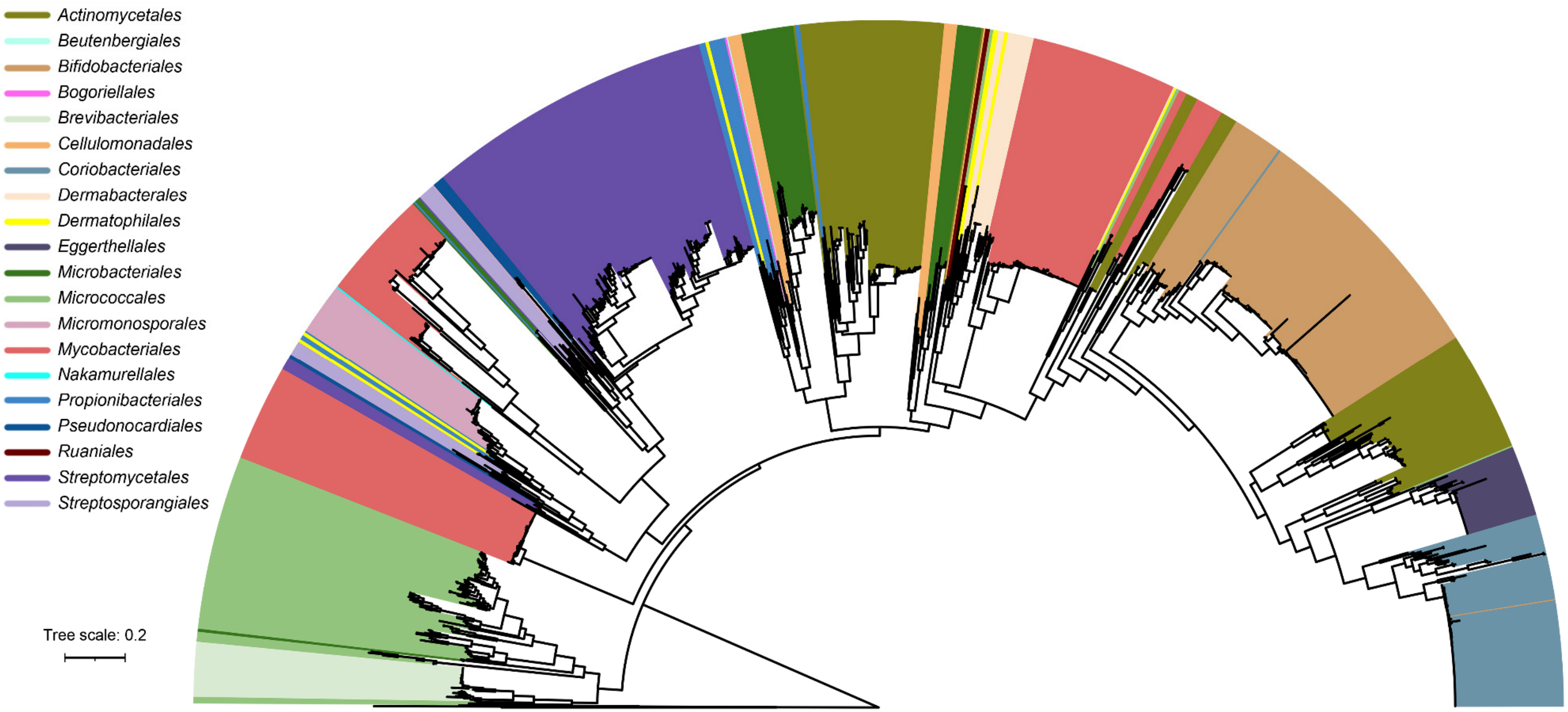


**Figure S3. Phylogeny of the concatenated NanA-K-E proteins of 1,290 actinobacterial strains.** The tree was produced by FastTree. Colored branches indicate different orders. Scale bar indicates 20% sequence divergence.

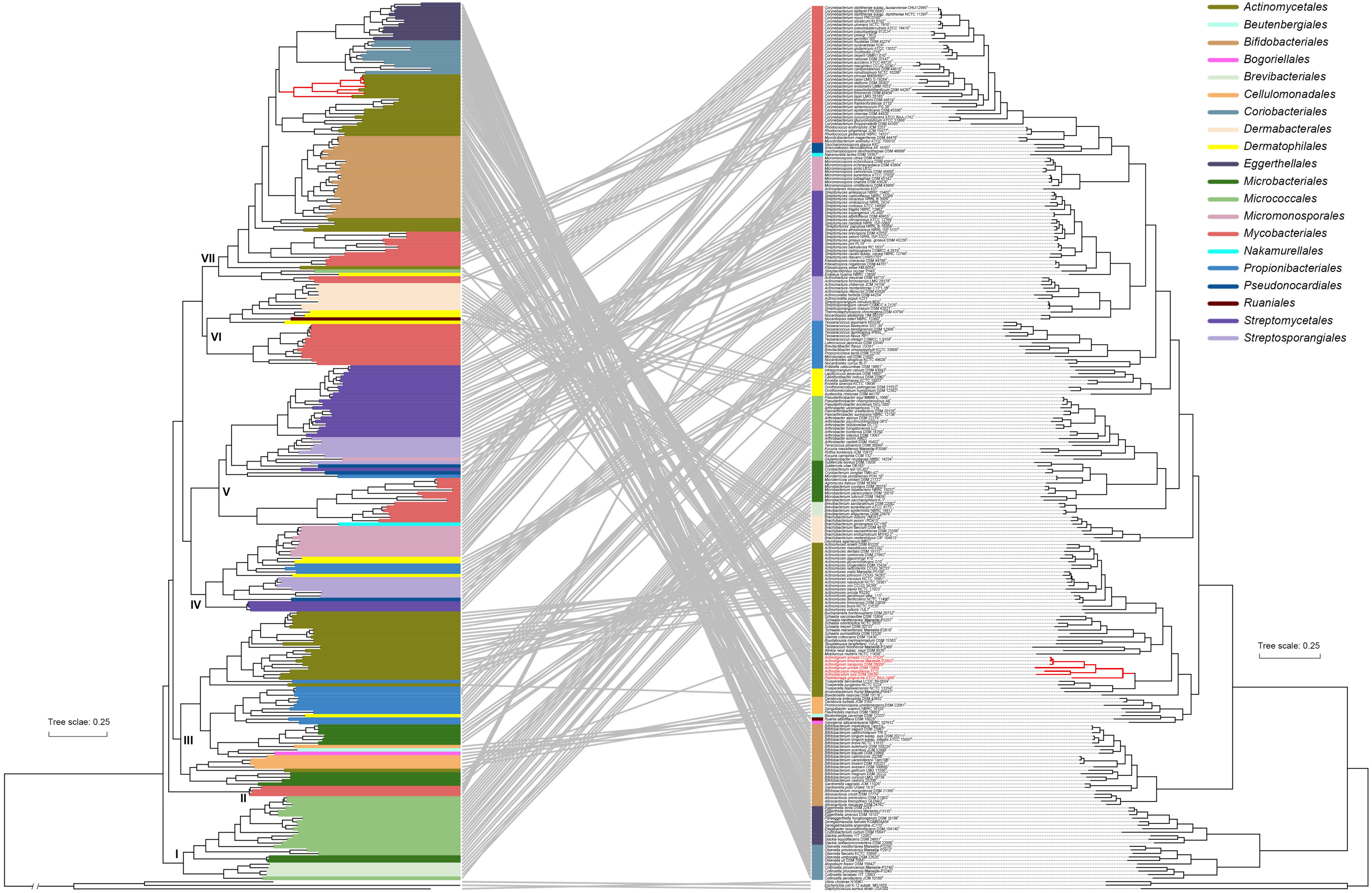


**Figure S4. Gene tree of the *nan* clusters in 255 actinobacterial species (left) compared to the corresponding species tree (right).** Both trees were produced by using FastTree. The gene tree is based on the concatenated NanA/K/E proteins, and the species tree is based on 138 universally conserved proteins of *Actinobacteria*. Gray lines show the comparisons of the positions of organisms on the two trees. Branch with the same topological structure in the two trees mentioned in the main text is marked in red. Different colors indicate different orders. “I” to “VII” indicates genetic clades of the gene tree. Scale bar indicates 25% sequence divergence.

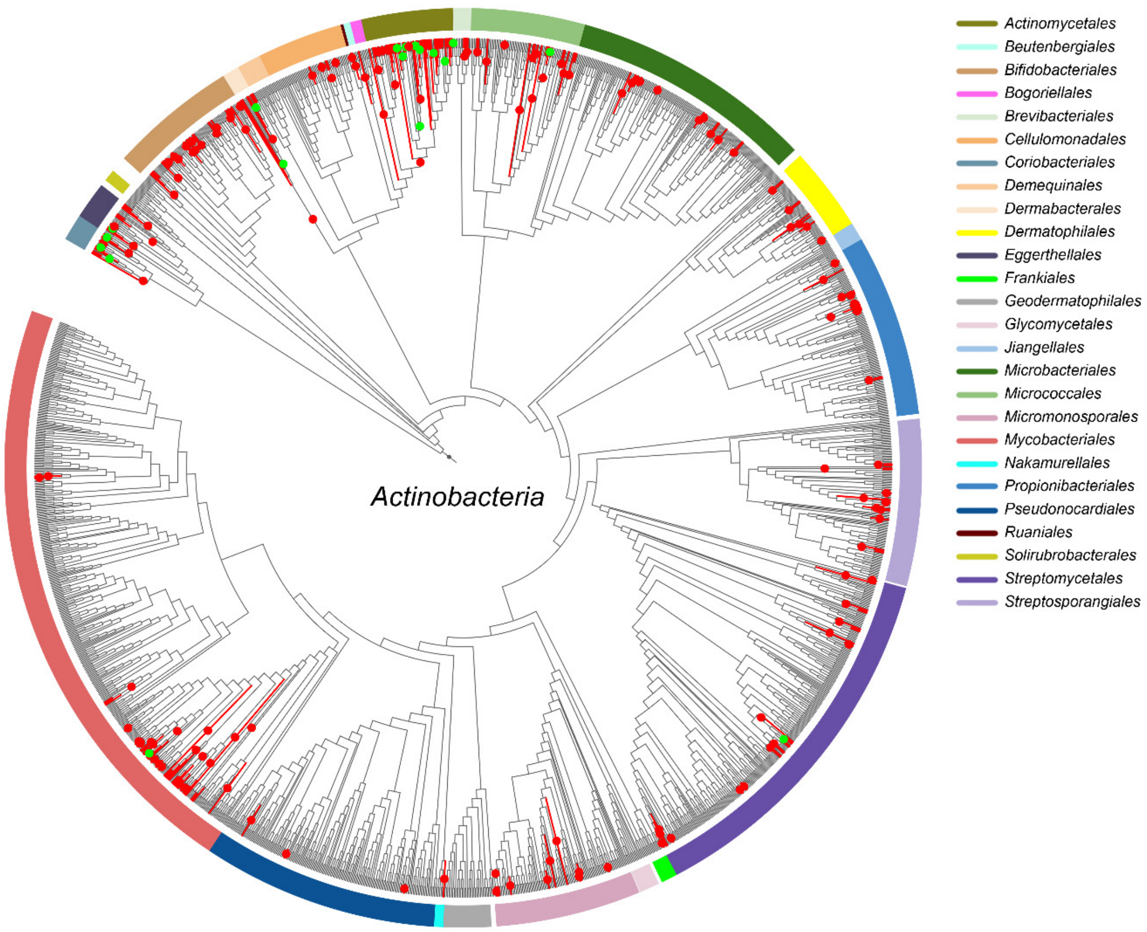


**Figure S5. Gene gain and loss events of the *nan* clusters during the evolution of actinobacterial species.** The tree is based on 138 universally conserved proteins of 1,969 type strains of the phylum *Actinobacteria*, and the branch lengths are ignored. Species with the *nan* clusters are marked as red at corresponding branches, and colored stripes surrounding the tree indicate different orders. Gain and loss events of the *nan* clusters are marked as dots in red and green, respectively, at nodes of the tree.

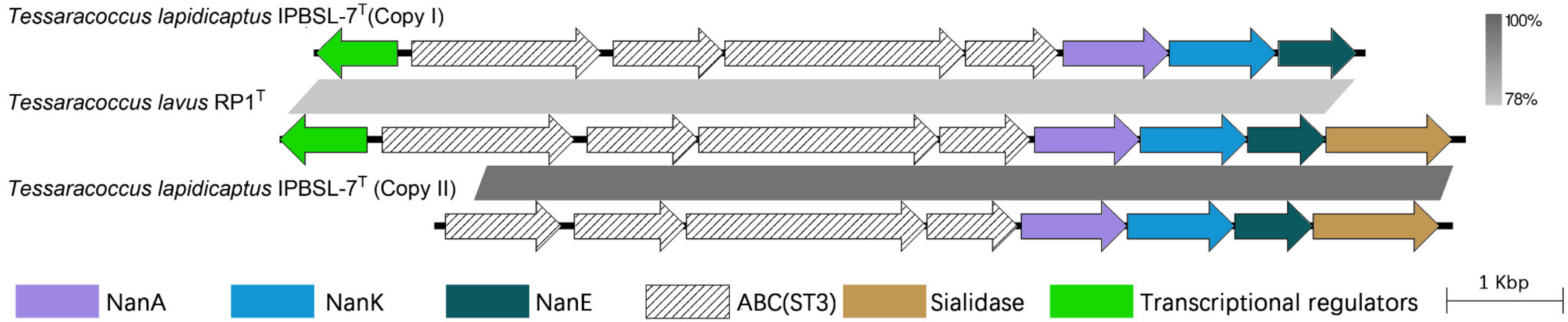


**Figure S6. Comparison of the genetic organization of the regions containing *nan* clusters in two *Tessaracoccus* species.** The figure was obtained by EasyFig, where gray-scale bars represent regions of shared similarity according to BLASTN. ABC, ABC transporter. ST, sialic acid transporter.
