# Supplementary material for: Distribution and Evolutionary History of Sialic Acid Catabolism in the Phylum Actinobacteria
Source: Microbiol Spectr. 2022 Jan 12;10(1):e02380-21. doi: 10.1128/spectrum.02380-21 (PMC8754123; doi:10.1128/spectrum.02380-21)
Supplement: Reviewer comments [file reviewer-comments.pdf]

AMERICAN SOCIETY FOR MICROBIOLOGY | Microbiology Spectrum

Distribution and evolutionary history of sialic acid catabolism in the phylum Actinobacteria

Yisong Li and Ying Huang

Corresponding Author(s): Ying Huang, Institute of Microbiology, Chinese Academy of Sciences

Review Timeline:

Submission Date:	November 26, 2021
Accepted:	December 9, 2021

Editor: Beile Gao

Reviewer(s): The reviewers have opted to remain anonymous.

Transaction Report:

(Note: With the exception of the correction of typographical or spelling errors that could be a source of ambiguity, letters and reports are not edited. The original formatting of letters and referee reports may not be reflected in this compilation.)

DOI: https://doi.org/10.1128/spectrum.02380-21

December 9, 2021

Prof. Ying Huang
Institute of Microbiology, Chinese Academy of Sciences
State Key Laboratory of Microbial Resources
NO.1 West Beichen Road, Chaoyang District
Beijing 100101
China

Re: Spectrum02380-21 (Distribution and evolutionary history of sialic acid catabolism in the phylum Actinobacteria)

Dear Prof. Ying Huang:

Your manuscript has been accepted, and I am forwarding it to the ASM Journals Department for publication. You will be notified when your proofs are ready to be viewed.

The ASM Journals program strives for constant improvement in our submission and publication process. Please tell us how we can improve your experience by taking this quick Author Survey.

As an open-access publication, Spectrum receives no financial support from paid subscriptions and depends on authors' prompt payment of publication fees as soon as their articles are accepted. You will be contacted separately about payment when the proofs are issued; please follow the instructions in that e-mail. Arrangements for payment must be made before your article is published. For a complete list of **Publication Fees**, including supplemental material costs, please visit ourwebsite.

Corresponding authors may join or renew ASM membership to obtain discounts on publication fees. Need to upgrade your membership level? Please contact Customer Service at Service@asmusa.org.

Thank you for submitting your paper to Spectrum.

Sincerely,

Beile Gao
Editor, Microbiology Spectrum

Journals Department
American Society for Microbiology
1752 N St., NW
Washington, DC 20036
E-mail: spectrum@asmusa.org

Supplemental Material 2: Accept
Supplemental Material 1: Accept
Supplemental Material 3: Accept
